# Secondhand smoke exposure toxicity accelerates age-related cardiac disease in old hamsters

**DOI:** 10.1186/1471-2261-14-195

**Published:** 2014-12-19

**Authors:** Jia-Ping Wu, Cheng-Hong Hsieh, Tsung-Jung Ho, Wei-Wen Kuo, Yu-Lan Yeh, Chien-Chung Lin, Chia-Hua Kuo, Chih-Yang Huang

**Affiliations:** Graduate Institute of Basic Medical Science, China Medical University, Taichung, Taiwan; Department of Health and Nutrition Biotechnology, Asia University, Taichung, Taiwan; School of Chinese Medicine, College of Chinese Medicine, China Medical University Beigang Hospital, Taichung, Taiwan; Chinese Medicine Department, China Medical University Beigang Hospital, Beigang, Taiwan; Department of Biological Science and Technology, China Medical University, Taichung, Taiwan; Department of Pathology, Changhua Christian Hospital, Changhua, Taiwan; Department of Medical Technology, Jen-Teh Junior College of Medicine, Nursing and Management, Miaoli, Taiwan; Orthopedic Department, Armed Forces General Hospital, Taichung, Taiwan; Department of Sports Sciences, University of Taipei, Taipei, Taiwan; School of Chinese Medicine, China Medical University, Taichung, Taiwan

**Keywords:** Left ventricular hypertrophy, Secondhand smoke exposure, Aging, Pro-inflammatory response, Aged SHS exposure

## Abstract

**Background:**

Aging is associated with physiological or pathological left ventricular hypertrophy (LVH) cardiac changes. Secondhand smoke (SHS) exposure is associated with pathological LVH. The action mechanism in cardiac concentric hypertrophy from SHS exposure is understood, but the transition contributed from SHS exposure is not. To determine whether exposure to SHS has an impact on age-induced LVH we examined young and old hamsters that underwent SHS exposure in a chamber for 30 mins.

**Methods:**

Morphological and histological studies were then conducted using hematoxylin and eosin (H&E) and Masson’s trichrome staining. Echocardiographic analysis was used to determine left ventricular wall thickness and function. LVH related protein expression levels were detected by western blot analysis.

**Results:**

The results showed that both young and aged hamsters exposed to SHS exhibited increased heart weights and left ventricular weights, left ventricular posterior wall thickness and intraventricular septum systolic and diastolic pressure also increased. However, left ventricular function systolic and diastolic pressure deteriorated. H&E and Masson’s trichrome staining results showed LV papillary muscles were ruptured, resulting in lower cardiac function at the myocardial level. LV muscle fiber arrangement was disordered and collagen accumulation occurred. Concentric LVH related protein molecular markers increased only in young hamsters exposed to SHS. However, this declined with hamster age. By contrast, eccentric LVH related proteins increased in aging hamsters exposed the SHS. Pro-inflammatory proteins, IL-6, TNF-α, JAK1, STAT3, and SIRTI expression increased in aging hamsters exposed to SHS.

**Conclusions:**

We suggest that SHS exposure induces a pro-inflammatory response that results in concentric transition to aging eccentric LVH.

## Background

Secondhand smoke (SHS) exposure is a well known contributor to cardiovascular disease among non-smokers [1, 2]. Little known about how SHS exposure affects aging changes. The molecular mechanisms of cardiac aging exposed to SHS are still unclear, especially, the transition from concentric to eccentric left ventricular hypertrophy. This study attempts to determine whether SHS exposure leads the transition from concentric to eccentric left ventricular hypertrophy (LVH) using molecular signaling pathway regulation markers. SHS genotoxicity leading to cardiovascular diseases resulting in human pathological cardiac hypertrophy has been confirmed [3, 4]. Previous studies elucidated that cigarette SHS exposure among children [5, 6] might cause irreversible impairment in endothelium-dependent vasodilation, but the impact in old age is still unclear. The aging heart undergoes slowly progressive structural changes and functional decline with age [7, 8]. A number of pathological mechanisms may arise from the deleterious effects of SHS exposure [6]. The physiologic changes in the aging cardiac include left ventricular hypertrophy, increased cardiac fibrosis and valvular degeneration [9, 10]. Aging changes in the elderly heart are associated with physiological and pathological LV hypertrophy. Aging heart changes can produce cardiac diseases, including coronary arteries [11], myocardial infarction, cardiac valves, aortic regurgitation [12, 13]. Without a doubt, LV hypertrophy is related to changes in cardiac morphology including increased myocyte size [14], increased left ventricular wall thickness and decreased fiber density [15, 16]. Once the aging heart experiences extended elevated workload, the left ventricle becomes deficient in pumping adequate blood. Thus, pathological aging related LV hypertrophy [17] may be associated with cardiovascular disease [18]. The physiological aging associated changes [19] in the heart cannot be reversed and deteriorate in function. The transition between LV concentric hypertrophy and LV eccentric hypertrophy is our interest.

## Methods

### Animals

We purchased male hamsters at ages 6 weeks and 72 weeks from the National Science Council Animal Center. The animals were group-housed six per cage in an animal room. The experimental protocols were conducted in accordance with committee approved animal care and experimental guidelines from the Taiwan Society for Laboratory Animals Sciences. Male hamsters were used in these studies. Only male hamsters are used to reduce the potential for variability from gender-related differences in cardiac aging. All the animal handling protocols were reviewed and approved by the Institutional Review Board (IRB), Animal care and use committee of the China Medical University, Taichung, Taiwan (ROC).

### Experimental design and secondhand smoke (SHS) exposures

Young and aged groups were divided into two subgroups for secondhand smoke (SHS) exposure. (1) control; hamsters were not exposed to secondhand cigarette smoke. (2) SHS exposure; hamsters exposed to cigarette secondhand smoke. The four groups of hamsters were subjected to experiments as follows: MYC; male young control, MYS; male young SHS exposure, MOC; male old control, MOS; male old SHS exposure. Hamsters were placed in an exposure chamber and then exposed to 10 cigarettes for 30 min, 4 weeks [19].

### Hematoxylin-eosin and Masson’s trichrome staining

To assess the left ventricle cross-sectional area and extracellular space, the cross sections were stained with hematoxylin and eosin and incubated for 5 min. Part of the left ventricular cross section was stained with Masson’s trichrome to detect collagen accumulation. The stained sections were then rinsed with PBS and air dried before mounting. After gently rinsing with water, the slides were dehydrated through a graded alcohol series for 15 min, cleaned in xylene and then covered with a slip.

### Left ventricular tissue collection and extraction

After the animal was sacrificed, the atrium and right ventricle were removed. The left ventricle was rinsed in normal saline and then weighed for left ventricular mass. Left ventricular tissue extracts were obtained at a concentration of 0.1 g tissue/mL PBS by homogenizing. The homogenates were then placed and centrifuged at 12,000 rpm for 30 mins.

### Immunohistochemistry analysis

The left ventricular samples from young and old hamsters were fixed and embedded in paraffin. Ten micrometer thick tissue sections were cut and then dewaxed and rehydrated. Slides were blocked in 0.1% bovine serum albumin with PBS buffer for 30 mins at room temperature and incubated with mouse SIRT1 monoclonal antibody in PBS for 2 hrs. After washing, sections were incubated for 30 mins with secondary antibody. After 5 mins washing, peroxidase was developed using diaminobenzidine chromogen (DAB) diluted in H_2_O_2_ buffer.

### Echocardiography

Hamsters were anesthetized with an intraperitoneal ketamine-xylazine-atropine mixture. We used a commercially available echocardiography system equipped with a 14 MHz liner transducer. Transthoracic echocardiography was performed on young control, old control and 2 or 4 weeks SHS exposure treated in young and old hamsters, using a HDI-5000 ultrasound machine [20]. The following parameters were measured and calculated using the M-mode image: left ventricular posterior at diastolic (LVPWd) and systolic (LVPWs) wall thickness, intraventricular septal at diastolic (IVSd) and systolic (IVSs), fractional shortening (FS%) and ejection fractional (EF%). All data were transferred online to computer for subsequent analysis.

### Western blotting analysis

Left ventricular samples (1 ug) were homogenized for 5 min and centrifuged at 8,000 g for 20 min. Proteins were electrophoresed by 10% polyacrylamide gels at 110 V for 90 min, and then transferred to PVDF paper at 100 mA for 2.5 hr. Incubated PVDF members in 1% BSA blocking buffer for 1 hr at room temperature. Polyclonal antibodies, ANP, BNP, p-MEK1, MEK1, p-ERK1/2, ERK1/2, GATA4, IL-6, TNF α, JAK1, STAT3, Calcineurin, p-NFAT, NFAT, p-MEK5, MEK5, p-ERK5 and ERK5 were incubated at room temperature for 2 hr. The immunoblots were washed three times in 5 ml and then incubated in the second antibody solution containing anti-rabbit, or anti-goat, or anti-mouse IgG horseradish peroxidase for 1 hr. Color development was presented in ECL.

### Statistical analysis

All data were assessed as the mean ± SD. The experimental results two-way ANOVA analysis was used to assess male young control (MYC), male young SHS exposure (MYS), male old control (MOC), and male old SHS exposure (MOS) groups. *p < 0.05, **p < 0.01, significant difference compared with male young control (MYC). ^#^p < 0.05, ^##^p < 0.01, significant difference compared with male old control (MOC).

## Results

### Heart and left ventricular characteristics

Table 1 presents the heart and left ventricular (LV) characteristics of male young control (MYC), male young SHS exposure (MYS), male old control (MOC) and male old SHS exposure (MOS) hamsters. Hamsters were placed in an exposure chamber and exposed to cigarettes daily for 4 weeks. We obtained four groups of heart and left ventricular (LV) morphology characteristics. The whole body weight in the MYS group was increased, whereas body weight was decreased in the MOC group compared with the MYC group. The whole heart (HW) weights in MYC, MYS, MOC and MOS groups were 0.46 ± 0.01, 0.52 ± 0.01 (p < 0.05), 0.60 ± 0.03 (p < 0.01) and 0.63 ± 0.03 g (p < 0.01), respectively, when compared with the MYC group. The left ventricular (LV) weights in MYC, MYS, MOC and MOS groups were 0.31 ± 0.01, 0.39 ± 0.01 (p < 0.05), 0.42 ± 0.03 (p < 0.01) and 0.53 ± 0.03 g (p < 0.01), compared with the MYC group. HW and LV weight in old and old SHS exposure were 0.53 ± 0.01 and 0.63 ± 0.03 g, respectively, when compared with the old group. The results from a two-way ANOVA suggested that the MYS, MOC and MOS groups tended toward hypertrophy (Table 1). The HW/BW ratio in MYC, MYS, MOC and MOS groups were 3.59 ± 0.10, 3.88 ± 0.07, 4.45 ± 0.15 (p < 0.01) and 5.85 ± 0.38 mg/g (p < 0.01), respectively, compared with the MYC group, and no significant difference was observed between MYS and MYC group. That is because of increased body weight in the young adult SHS exposure. Therefore, we could not find HW/BW and LV/BW ratios changes in the MYS group. However, we also observed the HW/BW and LV/BW ratios in aged SHS exposure groups were higher than those in the old control. That is because of decreased body weight observed in the aged SHS exposure group, compared to the other groups. This may be because of aging or toxic smoke effect. Interestingly, we examined the HW/tibial and LV/tibial ratios, we found the HW/tibial and LV/tibial ratios in MYS, MOC and MOS groups all increased. The HW/tibial ratio in MYS, MOC and MOS groups were 19.14 ± 0.36 (p < 0.05), 19.84 ± 0.36 (p < 0.01), 24.30 ± 0.70 (p < 0.01) mg/mm, respectively, when compared to the MYC group. The LV/tibial and HW/tibial ratios have the same increased and trend left ventricular hypertrophy.

**Table 1:** **Morphology of the left ventricle in male young control (MYC), male young SHS exposure (MYS), male old control (MOC) and male old SHS exposure (MOS) hamsters**

	Young		Old	
	MYC	MYS	MOC	MOS
Animal number (N)	6	6	6	6
BW (g)	132.54 ± 4.61	145.67 ± 3.42*	130.26 ± 6.04	127.59 ± 3.52*^♦#^
HW (g)	0.46 ± 0.01	0.52 ± 0.01*	0.60 ± 0.03**^♦^	0.63 ± 0.03**^♦♦#^
LV (g)	0.31 ± 0.01	0.39 ± 0.01	0.42 ± 0.01**^♦^	0.53 ± 0.01**^♦♦#^
HW/BW (mg/g)	3.59 ± 0.10	3.88 ± 0.07	4.45 ± 0.15**^♦^	5.85 ± 0.38**^♦♦#^
LV/BW (mg/g)	2.44 ± 0.05	2.67 ± 0.11	3.27 ± 0.05**^♦^	4.01 ± 0.20**^♦♦#^
HW/tibia (mg/mm)	16.21 ± 0.23	19.14 ± 0.36	19.84 ± 0.36**^♦^	24.30 ± 0.70**^♦♦#^
LV/tibia (mg/mm)	11.12 ± 0.25	13.66 ± 0.47	14.73 ± 0.20**^♦^	18.48 ± 0.48**^♦♦#^

Values of represent as mean ± SEM. BW, body weight; HW, whole heart weight; LV, left ventricle; HW/BW, ratio of whole heart weight; LV/BW, ratio of left ventricular weight to body weight; HW/tibia, ratio of whole heart weight to tibia length; LV/tibia, ratio of left ventricular to tibia length. *p < 0.05, **p < 0.01 significant difference vs MYC. ^♦^p < 0.05, ^♦♦^p < 0.01 siginificant difference vs MYS. ^#^p < 0.05, ^##^p < 0.01 significant difference from MOC.

### The SHS exposure effect on cardiac function in young and old hamsters

To identify whether the combination of SHS exposure and aging effects represent an adaptation of the heart or an indication of left ventricular dysfunction has been a matter of controversy. On the one hand, echocardiography is imaging method in the assessment of cardiac (Tables 2 and 3). Summarizes the echocardiographic characteristics in all groups. LVH was present in MYS, MOC and MOS hamsters groups. No significant differences at baseline systolic and diastolic in all young groups. No significant differences in left ventricular posterior wall thickness (LVPWd) and intraventricular septal (IVSd) at baseline diastolic in all aging groups were found. Surprisingly, we observed left ventricular posterior wall thickness (LVPWs) and intraventricular septal (IVSs) at baseline systemic were increased in aging and aging without SHS exposure hamsters groups. Because that aging leads to left ventricular posterior wall thickness at baseline systolic increased and intraventricular septal dysfunction is enhanced. We first discussed short-term 2 weeks SHS exposure, we found LVPWd and IVSs in MYS, MOC and MOS groups were all increased (all p < 0.05) when compared with MOS group, whereas LVPWs increased in MOC and MOS groups (p < 0.05), and IVSd was increased in MOS group (p < 0.05). However, when compared with the aging (MOC group), we observed LVPWd from 1.9 ± 0.1 increased to 2.0 ± 0.3 (p < 0.05), IVSd from 1.3 ± 0.1 increased to 1.5 ± 0.3 (p < 0.05), IVSs from 1.9 ± 0.1 increased to 2.0 ± 0.1 (p < 0.05). LVPWs exhibited no significant differences in aged exposure to SHS. Furthermore, long-term 4 weeks exposure to SHS exposure, we found LVPWd, LVPWs, IVSd and IVSs in MYS, MOC and MOS groups were all increased when compared with the MYC group (all p < 0.05). Interestingly, no significant difference in MOS group was found, when compared with the MOC group. It is possible with aging. There were no significant differences between aging and aged SHS exposure. However, Fraction Shortening (FS%) and Ejection Fraction (EF%) were progressive impairment in MYC, MYS, MOC and MOS groups at 2 weeks and 4 weeks SHS exposure (Table 3). LV function declines were observed. In fact, as Table 3 Shows, the systolic function of left ventricle in MOC and MOS groups at baseline trended toward lower EF% and FS% (p < 0.05). These data are strongly indicative of the elder hamster’s left ventricular dysfunction. Two or 4 weeks SHS exposure, LV function of EF (%) and FS (%) reduced in young and old hamsters. Indeed, the aging hamsters exposure to SHS were significantly lower than old hamsters (p < 0.01).

**Table 2:** **Changes characteristic of left ventricle in male young control (MYC), male young SHS exposure (MYS), male old control (MOC) and male old SHS exposure (MOS) hamsters**

		Unit	MYC	MYS	MOC	MOS
Animals			6	6	6	6
LVPWd	(baseline)	mm	0.9 ± 0.2	0.9 ± 0.2	1.0 ± 0.1	1.0 ± 0.4
LVPWd	(2 weeks)	mm	1.2 ± 0.1	1.9 ± 0.1*	1.9 ± 0.1*	2.0 ± 0.3*^#^
LVPWd	(4 weeks)	mm	1.5 ± 0.2	2.0 ± 0.7*	2.0 ± 0.1*	2.0 ± 0.2*
LVPWs	(baseline)	mm	1.7 ± 0.1	1.7 ± 0.7	1.7 ± 0.1	1.9 ± 0.3*
LVPWs	(2 weeks)	mm	1.7 ± 0.1	1.7 ± 0.3	2.0 ± 0.2*	2.0 ± 0.2*
LVPWs	(4 weeks)	mm	1.7 ± 0.1	2.0 ± 0.1*	2.0 ± 0.1*	2.0 ± 0.3*
IVSd	(baseline)	mm	1.0 ± 0.1	1.0 ± 0.1	1.0 ± 0.1	1.1 ± 0.1
IVSd	(2 weeks)	mm	1.3 ± 0.1	1.3 ± 0.1	1.3 ± 0.1	1.5 ± 0.3^*#^
IVSd	(4 weeks)	mm	1.5 ± 0.1	2.0 ± 0.3*	2.0 ± 0.2*	2.0 ± 0.3*
IVSs	(baseline)	mm	1.3 ± 0.1	1.4 ± 0.2	1.7 ± 0.3*	1.6 ± 0.2*
IVSs	(2 weeks)	mm	1.5 ± 0.2	1.9 ± 0.1*	1.9 ± 0.1*	2.0 ± 0.1^*#^
IVSs	(4 weeks)	mm	1.6 ± 0.2	2.1 ± 0.2*	2.0 ± 0.1*	2.1 ± 0.1*

Values are mean ± S.E. LVPWd, left ventricular posterior wall thickness at diastole; LVPWs, left ventricular posterior wall thickness at systolic; IVSd, Interventricular septum at diastolic; IVSs, Interventricular septum at systolic. *p < 0.05, **p < 0.01 siginificant difference vs MYC. ^#^p < 0.05, ^##^p < 0.01 significant differentce from MOC.

**Table 3:** **Left ventricular function**

		Young		Old	
	Unit	MYC	MYS	MOC	MOS
EF (baseline)	%	82.05 ± 5.48	72.91 ± 3.47	67.88 ± 8.03*	66.47 ± 6.26*
EF (2 weeks)	%	83.63 ± 1.20	67.33 ± 7.75*	62.12 ± 2.82*	58.33 ± 2.67**^#^
EF (4 weeks)	%	79.33 ± 1.84	66.33 ± 3.18*	62.30 ± 2.66*	40.14 ± 8.57**^#^
FS (baseline)	%	45.99 ± 5.46	40.51 ± 2.85	33.15 ± 5.50*	31.49 ± 4.71*
FS (2 weeks)	%	45.28 ± 2.28	32.33 ± 5.24*	31.90 ± 1.64*	27.33 ± 1.67**^#^
FS (4 weeks)	%	41.28 ± 2.48	32.05 ± 2.52*	27.75 ± 2.27*	17.67 ± 4.67**^#^

Values are mean ± S.E. FS, fractional shortening, EF, ejection fractional. *p < 0.05, **p < 0.01 significant difference vs MYC. ^#^p < 0.05, ^##^p < 0.01 siginificant differentce from MOC.

### Changes in left ventricular architecture in male young control, male young SHS exposure, male old control and male old SHS exposure hamsters

**Figure 1:**
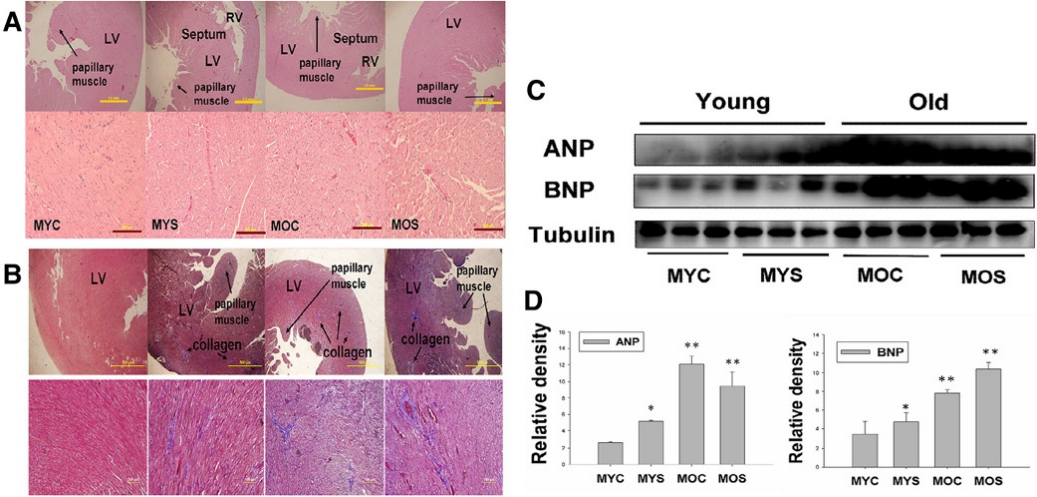
**Representative histological cross sections of the left ventricle (LV) stained with hematoxylin-eosin stain and Masson’s trichrome staining.** Substantial left ventricles remodeling in old age heart and secondhand smoke exposure. **(A)** Morphological features of the left ventricle of hematoxylin-eosin stained. Morphometric samples are at x100 magnification (up-panel). Morphometric samples are at x400 magnification) (down-panel). The arrow points to the papillary muscle. **(B)** Histological examination of LV fibrosis by Masson’s trichrome staining. Morphological features of left ventricular hematoxylin-eosin stained. Morphometric samples are at x100 magnification (up-panel). Morphometric samples are at x400 magnification) (down-panel). The long arrow points to the papillary muscle, short arrow points to collagen. **(C)** western blotting for ANP and BNP protein in MYC, MYS, MOC and MOS groups from the indicated left ventricular extracts. **(D)**. Statistical analysis ANP and BNP protein expression levels in MYC, MYS, MOC and MOS groups. Data are means ± SD. *P < 0.05, **P < 0.01 significantly statistical differences vs. MYC group (two-way ANOVA). MYC; male young control, MYS; male young SHS exposure, MOC; male old control, MOS; male old SHS exposure.

Hamsters showed tissue architectures and histopathologic of LV hypertrophy in MYC, MYS, MOC and MOS groups on hematoxylin-eosin staining (H&E) (Figure 1A) and LV fibrosis by Masson’s trichrome staining (Figure 1B). According to the results, we found LV papillary muscle rupture, LV chamber narrow, LV papillary muscle space increases and observed LV muscle fibers dissociation, arrangement disarray, relaxation, and interstitial spaces broad in MYS, MOC and MOS groups using H&E staining (Figure 1A and B). Histological examination also revealed the extent of LVH. Morphometric samples are at x100 magnification (Figure 1A, up-panel) and x400 magnification (Figure 1A, down-panel). In Masson’s trichrome staining, we observed blue collagen accumulation of LV fibrosis in the MYS, MOC and MOS groups. Morphometric samples are at x100 magnification (Figure 1B, up-panel) and x400 magnification (Figure 1B, down-panel). This finding supported by data Table 1 and Table 2 showing aging and SHS exposure trended to LVH and fibrosis. The molecular mechanisms of cardiac hypertrophy markers, ANP and BNP protein expression were detected by western blotting analysis. To determine whether left ventricular will become hypertrophy Figure 1C shows that ANP and BNP increased in MYS, MOC and MOS groups, when compared with the MYC group. There was no significant difference between aging and aging SHS exposure.

### Molecular characterization of MEK1-ERK1/2-GATA4 signaling pathway in regulation of concentric left ventricular hypertrophy

**Figure 2:**
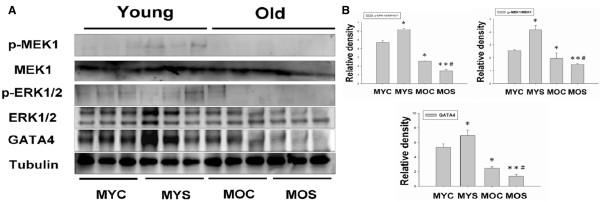
**MEK1-ERK1/2-GATA4 signaling pathway in male young exposure to SHS promotes left ventricular concentric hypertrophy. (A)**. Western blot analysis of p-MEK1, MEK 1, p-ERK1/2, ERK1/2 and GATA4 protein expression in the MYC, MYS, MOC and MOS groups. **(B)**. Quantification of densitometry analysis of protein expression levels. Statistical analysis of the p-MEK1/MEK1 and p-ERK1/2/ERK1/2 ratios and GATA4 densitometry in MYC, MYS, MOC and MOS groups. All data are presented as means ± SD. *p < 0.05, **p < 0.01 significantly statistical differences compared with male young control (MYC). *p < 0.05, **p < 0.01 significantly statistical differences compared with male old control (MOC).

MEK1-ERK1/2-GATA4 signaling pathway regulates the left ventricle balance between concentric and eccentric growth [20]. To explore whether MEK1-ERK1/2-GATA4 signaling pathway activation induced concentric left ventricular hypertrophy, inhibition of this pathway resulted in eccentric hypertrophy in aging and aged SHS exposure hamsters. The MEK1-ERK1/2-GATA4 signaling pathway regulates concentric cardiac hypertrophy. To determine the MEK1-ERK1/2-GATA4 signaling pathway as a potential regulator of concentric hypertrophy in MYS, MOC and MOS groups we examined p-MEK1, MEK1, p-ERK1/2, ERK1/2 and GATA4 protein expression levels using western blotting analysis. As Figure 2 shows, MEK1 and its active form of p-MEK1 were increased in the MYS group, but decreased in the MOC and MOS groups (Figure 2A). The results showed a two-way ANOVA statistical analysis and then student’s *t*-test used in the comparison between MOC and MOS group. The ratio of p-MEK1 to MEK1 was increased in MYS group (p < 0.05), but decreased in MOC (p < 0.05) and MOS groups (p < 0.01), when compared with MYC. However, significant differences between the MOC and MOS groups (p < 0.05) were found. We found ERK1/2, p-ERK1/2, and transcription factor, GATA4, have the same results with MEK1 expression trend to MYS group increase, but a trend toward reduction in the MOC and MOS groups (Figure 2A). When compared with MOC group, we found MOS group protein expression was significantly lower than that for the MOC group. Protein expression levels were quantified using analysis of variance (ANOVA) to assess all experimental groups. GATA 4 relative density and ratio of p-ERK1/2 to ERK1/2 were increased in MYS group (p < 0.05), and in the MOC (p < 0.05) and MOS groups (p < 0.01) decreased, when compared with MYC group (Figure 2B). When compared with aging, we found MOS group was lower than MOC group (p < 0.05) (Figure 2B). The results suggest only MYS group tended to LV concentric hypertrophy.

### Inflammatory response evidences linking secondhand smoke (SHS) exposure with pathological age-related diseases

**Figure 3:**
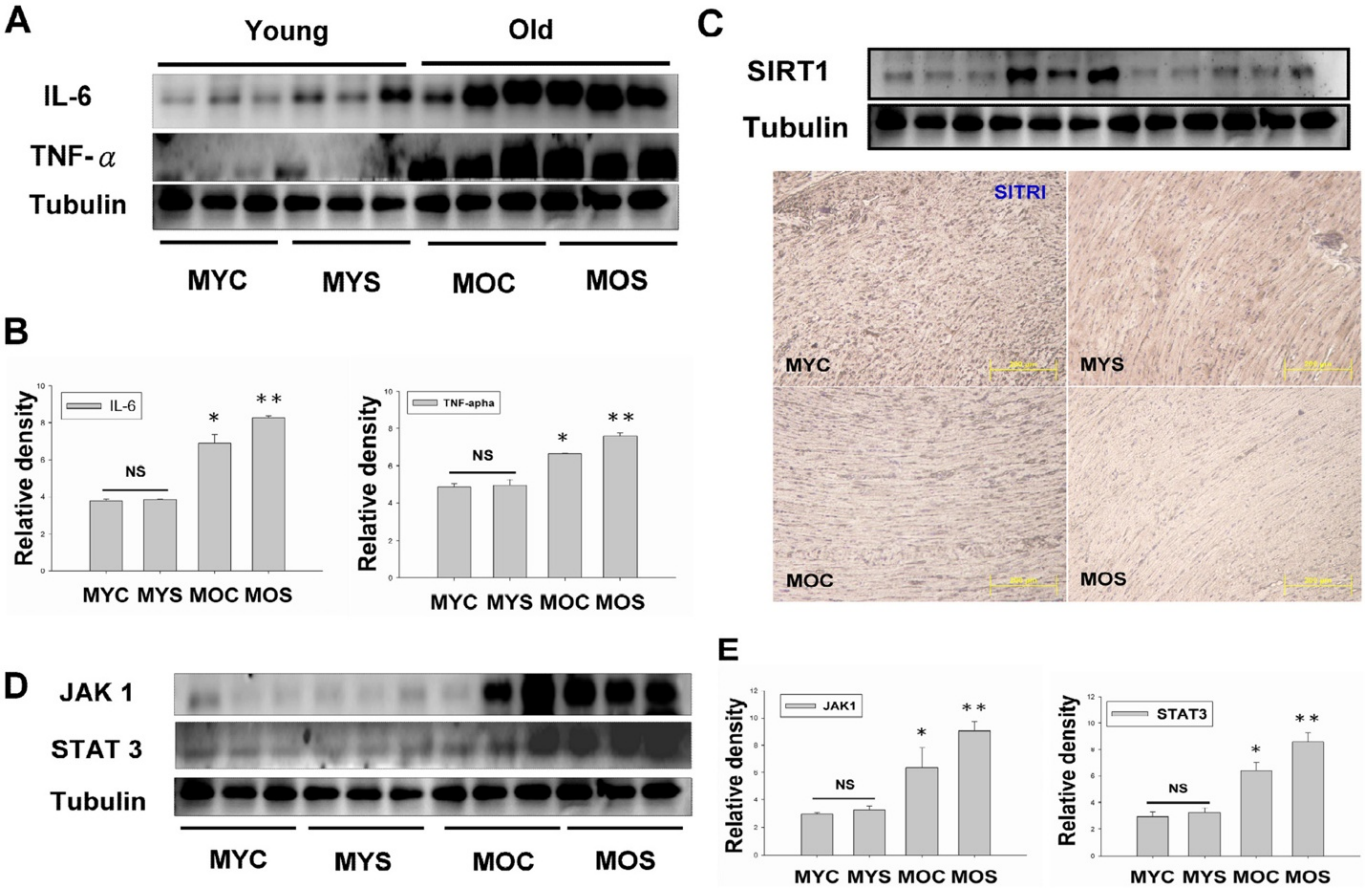
**Inflammatory cytokines, IL-6, TNFα, JAK1 and STAT3 mediated eccentric left ventricular hypertrophy in aging and aged SHS exposure hamsters. (A)**. Western blot analysis of Inflammatory protein, IL-6 and TNFα, expression in MYC, MYS, MOC and MOS groups. **(B)**. Quantification of densitometry analysis of IL-6 and TNFα protein expression levels. Statistical analysis of IL-6 and TNFα protein expression levels in MYC, MYS, MOC and MOS groups. All data are presented as means ± SD. *p < 0.05, **p < 0.01 significant statistical differences compared with male young control (MYC). **(C)**. Western blot and immunohistochemical analysis detection of SIRT1 in the four experimental groups were measured. **(D)**. Western blot analysis of cytokines, JAK1 and STAT3 protein expression in MYC, MYS, MOC and MOS groups. **(E)**. Quantification of densitometry analysis of JAK1 and STAT3 protein expression levels. All data are presented as means ± SD. *p < 0.05, **p < 0.01 significant statistical differences compared with male young control (MYC).

Cytokines, TNF α, IL-6, JAK1 and STAT3 as markers of inflammation response may play a major role in the risk for cardiovascular disease, which is also recognized as essential mediator of pathological aging. To determine whether inflammatory processes are linked to the involvement of inflammation in the SHS exposure-related concentric LV hypertrophy transition to age-related eccentric LV hypertrophy pathogenesis. According Figure 3A,C and D, TNF α, IL-6, JAK1, STAT3 and SIRT1 protein expression by western blotting analysis were increased in MOC (p < 0.05) and MOS groups (p < 0.01), when compared with the MYC group (Figure 3). Interestingly, statistical analysis observed no significant difference in the MYS group when compared with the MYC group. However, compared with the MOC group, the MOS group showed no significant difference in changes compared with the MOC group. According to immunohistochemistry results showed SIRT 1 expression in the MYC, MYS, MOC and MOS groups. We observed SIRT 1 expression was as disclosed at immunohistochemistry in MYC and MYS groups. However, old group expression was weaker including aged SHS exposure group (Figure 3C). Experimental evidences inflammatory response leads aging tended toward pathological age-related LV hypertrophy.

### Calcineurin/NFAT-MEK5-ERK5 signaling pathway induced aging related pathological left ventricular eccentric hypertrophy

**Figure 4:**
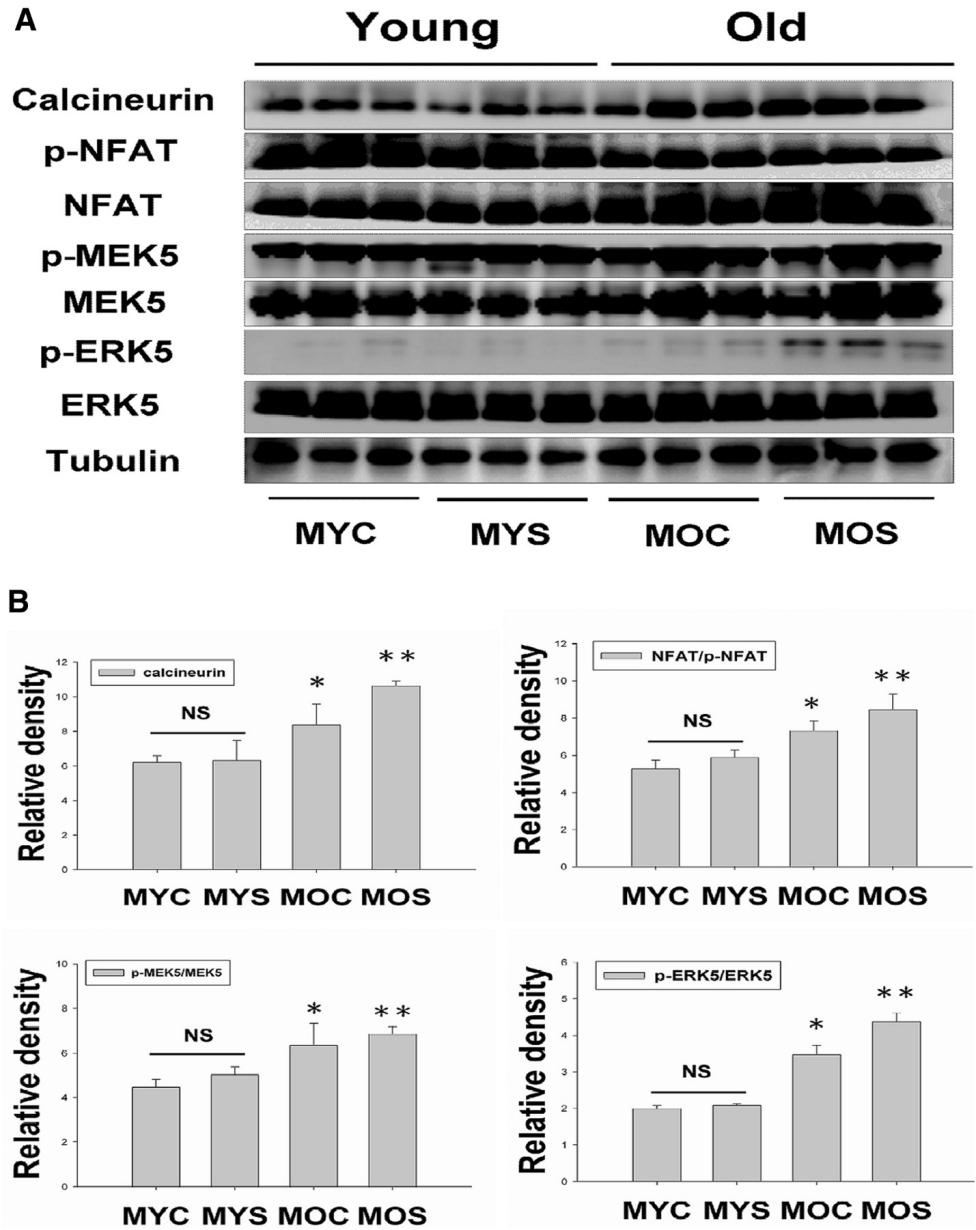
**Stimulation of calcineurin-NFAT, p-MEK5-MEK5, and p-ERK5-ERK5 activation promote eccentric left ventricular hypertrophy in aging and aged SHS exposure. (A)**. Protein expression levels of calcineurin, NFAT, p-NFAT, p-MEK5, MEK5, p-ERK5 and ERK5 in the MYC, MYS, MOC and MOS groups were measured by western blotting analysis. **(B)**. Statistical analysis of calcineurin densitometry and NFAT/p-NFAT, p-MEK5/MEK5, and p-ERK5/ERK5 ratios in MYC, MYS, MOC and MOS groups. All data are presented as means ± SD. *p < 0.05, **p < 0.01 significant statistical differences compared with male young control (MYC).

NFAT is the Ca^2+_^ dependent calcineurin which was identified as an eccentric hypertrophic signaling molecule in the myocardium in pathological cardiac hypertrophy. We discuss recent findings related continued activation of calcineurin/NFAT whether throughout the aging included eccentric cardiac hypertrophy. Our data showing calcineurin/NFAT protein expression increases was to maintain the hypertrophy profile of the left ventricle in the aging, but calcineurin/NFAT activation was also increased in the aged SHS exposure (Figure 4A). Interestingly, calcineurin and NFAT expression did not found increased in MYS group, but could observe the p-NFAT expression increased (Figure 4A). Quantitative and statistic analysis results, we found calcineurin protein expression was increased in MOC (p < 0.05) and MOS (p < 0.01) groups, when compared with MYC group. No significant difference between MYC and MYS groups (Figure 4B). The NFAT/p-NFAT ratio in the MOC and MOS groups were increased (p < 0.05 and p < 0.01, respectively), when compared with the MYC group. ERK5 is activated by the upstream MEK 5 kinase in responses to LV eccentric hypertrophy. From a two-way ANOVA analysis, we found the p-MEK5/MEK and p-ERK5/ERK5 ratios in MOC (p < 0.05) and MOS (p < 0.01) groups were increased compared with MYC group (Figure 4B). We suggest that aging and aged SHS exposure trended toward eccentric LVH.

## Conclusions

**Figure 5:**
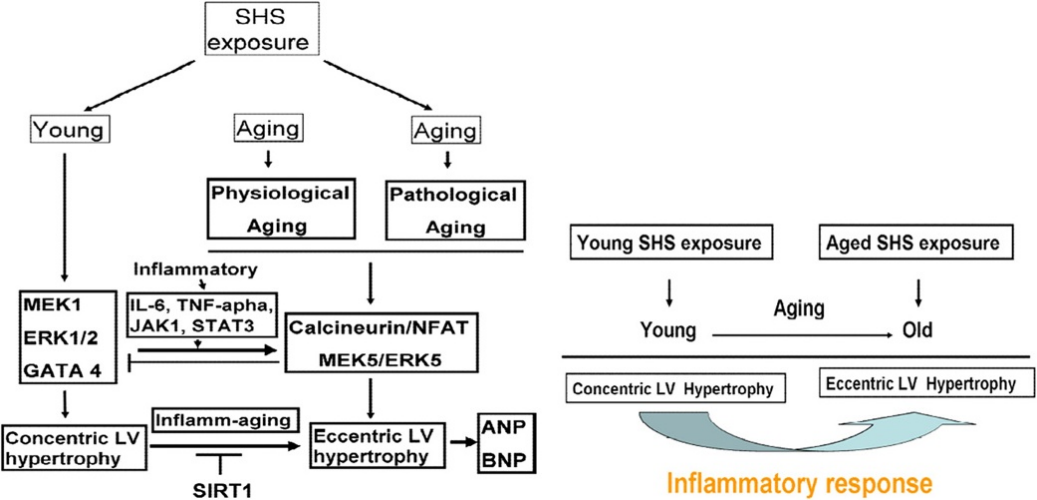
**The underlying of pathophysiological mechanisms related to aging exposure to SHS are depicted.** Inflamm-aging changes lead to concentric LV hypertrophy transfer to eccentric LV hypertrophy.

The left ventricular hypertrophy (LVH) phase during adaptive stress and pressure overload is the major reason individual myocytes grew into concentric or eccentric LVH [21, 22]. The aging effects on cardiovascular disease are similar to SHS exposure [23, 24]. To determine secondhand smoke (SHS) exposure transition to concentric LVH to aging- induced eccentric, SHS exposure and aging changed the left ventricular morphology and reduced heart function at systolic and diastolic (Tables 1, 2, and 3). We observed that left ventricular mass increases and left ventricular wall thickness increased. Given the above evidence which can be preliminary determined as cardiac or left ventricular hypertrophy, according to two-dimensional targeted M-mode echocardiogram results, we suggest that SHS exposure and aging lead to LV pathological hypertrophy. Recent studies have advanced the notion that SHS exposure would impair endothelial function, and which was a time- and concentration-dependent main [25, 26], in fact, the aged SHS exposure-induced the coronary arteries is easily developed cardiac valves and coronary stenosis. Nevertheless, aging is a physiological response which is an irreversible system in our life [27], but in a bad environment such as alcohol, smoke and SHS exposure, which will lead this physiological aging transfer pathological aging. From H&E staining and fetal genes expression, we observed cross-section of pathological LV hypertrophy and fibrosis as well as commonly associated with up regulation of ANP and BNP protein expression by western blotting (Figure 1). We demonstrate that LV hypertrophy does occur in the normal elderly who do not have heart disease. A previous study demonstrated that the normal aging process affects clinical heart disease in ways such as hypertensives, implying more pronounced reductions in cardiac and vascular compliance, because aortic valve calcium and mitral valve annular calcium appeared [28]. However, it is debatable whether the aged SHS exposure resulted in LV pathological hypertrophy which maybe occur eccentric. Goldenburg had clearly demonstrated that it is this property of the hypertrophied heart which the muscle fibres are increased in size, the nuclei are larger than normal, and there is generally present a hyperplasia. In other words, the expression of the overwork heart showed an absolute and relative increase in the size of the heart, and the relative weights of the heart and body. A left ventricular hypertrophy should place an predominant role resulted from hypertension in the systemic circulation. The fact, we justified bear out this assumption that the principal factors in the production of cardiac hypertrophy is an aortic insufficiency. Aortic insufficiency resulted in the blood backflow during cardiac diastolic condition. Aging is considered as a major risk factor cardiovascular diseases. We conclude SHS exposure will be exacerbated age- related left ventricular hypertrophy. Research further elucidating the underlying development of pathological mechanisms and potential countermeasures. These explore knowledge may influence therapeutic strategies for the treatment of cardiovascular disease in old age [28, 29]. Accumulated all evidence indicate that MEK1-ERK1/2-GATA4 signaling pathway induced concentric LVH in young SHS exposure, but not find aging and aged SHS exposure, whereas calcineurin/NFAT-MEK5-ERK5 signaling pathway activation-induced eccentric LVH was observed. One possibility is that transcription factors such as NFAT and GATA4 [30] are up-regulated in response to calcium-independent pathway of hypertrophy. Altered intracellular calcium is beyond the scope of the aging [31]. The other reason may be two signaling pathways have crosstalk to each other. A further possibility is SHS exposure-induced inflammation response resulted in TNFα, IL-6, JAK1, STAT3 and SIRT1 protein expression increases only in aging hamsters (Figure 3). Low concentration and short-term SHS exposure time only affected aging hamsters. Therefore, we can make sure that inflammation occurred in aging. On the other hand, MEK1-ERK1/2-GATA4 signaling inhibited calcineurin/NFAT and MEK5/ERK5 signals, and which attenuate eccentric LVH response [32, 33]. Indeed, MEK1/ERK1/2 [34], MEK5/ERK5 [35], JAK1/STAT3 [36] and calcineurin/NFAT [37] are cooperatively regulated hypertrophy expression. Indeed, SHS exposure results in superimposed with aging effects. Only in aging has detrimental consequences, one possibility is that low dose and short time exposure to SHS could be explained [38, 39]. SIRT1 expression can ascertain the aging degree and suppressed inflammatory aging. The underlying of pathophysiological mechanisms related to aging and exposure to SHS are depicted in Figure 5. Based on the presented evidences, it is concluded that transient exposure to low dose SHS may cause significant adverse effects on the aging human body presenting an acute health hazard.
